# Association between Visual Acuity and Retinal Layer Metrics in Diabetics with and without Macular Edema

**DOI:** 10.1155/2018/1089043

**Published:** 2018-10-03

**Authors:** LakshmiPriya Rangaraju, Xuejuan Jiang, J. Jason McAnany, Michael R. Tan, Justin Wanek, Norman P. Blair, Jennifer I. Lim, Mahnaz Shahidi

**Affiliations:** ^1^Department of Ophthalmology and Visual Sciences, University of Illinois at Chicago, Chicago, IL, USA; ^2^Department of Ophthalmology, University of Southern California, Los Angeles, CA, USA

## Abstract

**Purpose:**

Diabetes is known to cause alterations in retinal microvasculature and tissue that progressively lead to visual impairment. Optical coherence tomography (OCT) is useful for assessment of total retinal thickening due to diabetic macular edema (DME). In the current study, we determined associations between visual acuity (VA) and retinal layer thickness, reflectance, and interface disruption derived from enface OCT images in subjects with and without DME.

**Materials and Methods:**

Best corrected VA was measured and high-density OCT volume scans were acquired in 149 diabetic subjects. A previously established image segmentation method identified retinal layer interfaces and locations of visually indiscernible (disrupted) interfaces. Enface thickness maps and reflectance images of the nerve fiber layer (NFL), combined ganglion cell and inner plexiform layer (GCLIPL), inner nuclear layer (INL), outer plexiform layer (OPL), outer nuclear layer (ONL), photoreceptor outer segment layer (OSL), and retinal pigment epithelium (RPE) were generated in the central macular subfield. The associations among VA and retinal layer metrics were determined by multivariate linear regressions after adjusting for covariates (age, sex, race, HbA1c, diabetes type, and duration) and correcting for multiple comparisons.

**Results:**

In DME subjects, increased GCLIPL and OPL thickness and decreased OSL thickness were associated with reduced VA. Furthermore, increased NFL reflectance and decreased OSL reflectance were associated with reduced VA. Additionally, increased areas of INL and ONL interface disruptions were associated with reduced VA. In subjects without DME, increased INL thickness was associated with reduced VA, whereas in subjects without DME but with previous antivascular endothelium growth factor treatment, thickening of OPL was associated with reduced VA.

**Conclusions:**

Alterations in retinal layer thickness and reflectance metrics derived from enface OCT images were associated with reduced VA with and without presence of DME, suggestive of their potential for monitoring development, progression, and treatment of DME.

## 1. Introduction

Diabetes is known to cause alterations in retinal microvasculature and tissue that progressively lead to visual impairment. Indeed, diabetic retinopathy (DR) is the leading cause of vision loss in working-age adults [1]. One consequence of DR is the development of diabetic macular edema (DME) due to the accumulation of fluid within the central retinal tissue, which is a major contributor towards vision loss [2]. Reduction in visual acuity (VA) with progression of DR based on fundus photography in individuals with DME has been established [3]. Optical coherence tomography (OCT) is currently the standard of clinical care for detecting abnormalities in retinal structure and quantifying the extent of retinal thickening due to DME [4]. Furthermore, high-resolution OCT can also quantify subtle retinal thickening not discernible on clinical examination in individuals with mild DME [5]. Additionally, previous studies have shown methods for 3D OCT imaging [6] and repeatable retinal layer thickness measurements in healthy and multiple sclerosis patients using commercially available OCT instruments (Cirrus HD-OCT and Spectralis SD-OCT) [7].

Although the total retinal thickness in the central subfield has been shown to be correlated with VA, the association was weaker than that of individual retinal layers [7, 8]. Specifically, thinning of the nerve fiber layer (NFL) in individuals with minimal or no DR and thickening of the inner nuclear layer (INL) and outer plexiform layer (OPL) in individuals with DME have been reported [9–15]. In addition to these changes, reduced VA has been correlated with thinning of the ganglion cell layer + inner plexiform layer (GCLIPL) and the photoreceptor outer segment layer (OSL) in subjects with and without DME [16, 17].

In addition to retinal thickness, alterations in retinal layer reflectance and interface have also been reported in DR. Specifically, in subjects with DME, reductions in photoreceptor outer segment length and disruptions of photoreceptor inner/outer segment junctions have been related to reduced VA [18, 19]. Additionally, inner retinal layer interface disruptions (visually indiscernible layer interfaces) and discontinuities in the inner segment/outer segment junction and in the external limiting membrane were associated with reduced VA [20–23]. However, most previous studies have examined retinal layer thickness, reflectance, and interface disruption from single OCT B-scans which limits localization of the spatial extent of retinal abnormalities. To better understand the spatial characteristics of retinal pathology, a method of retinal layer segmentation that generates three-dimensional outer retinal topography and reflectivity maps has been developed [24]. Although this approach provides more information than that obtained from single B-scans, the algorithms were based on images obtained using a spectral domain OCT prototype instrument. Using a commercially available OCT instrument, we have previously reported and validated methods for generating enface thickness maps and reflectance images of individual retinal layers from a high-density raster of OCT B-scan images and demonstrated alterations at different stages of DR [25–27]. In the current study, we identified individual retinal layers with thickness, reflectance, and interface disruption associated with VA in groups of DR subjects with and without DME.

## 2. Methods

### 2.1. Subjects

The research study was approved by an Institutional Review Board at the University of Illinois at Chicago and followed the tenets of the Declaration of Helsinki. Prior to enrollment, the study was explained to the subjects and informed consent was obtained. A total of 149 diabetic subjects participated in the study. All subjects underwent clinical examination by retinal specialists. Exclusion criteria were refractive error greater than 6 diopters of myopia, clinical diagnosis of glaucoma, age-related macular degeneration, retinal vascular occlusions or other conditions that could alter the anatomic integrity of the retina, history of intraocular surgery, cataract surgery performed less than 4 months prior to imaging, lens nuclear sclerosis score greater than 2+, or posterior subcapsular cataract concurrent with VA less than 20/20. One eye per subject was selected based on the exclusion criteria. If both eyes qualified, the eye with better image quality was included. Based on clinical examination by retina specialists, subjects' eyes were classified as no DR (NDR; *N* = 51), nonproliferative DR (NPDR; *N* = 59), or proliferative DR (PDR; *N* = 39). The subjects were categorized into two subgroups, DME and no-DME, based on central subfield thickness (CST) being greater than 320 *μ*m (males) and 304 *μ*m (females) [28]. Twenty-eight subjects (NDR = 2; NPDR = 16; PDR = 10) had DME and 121 did not have DME at the time of imaging. Twenty-one of the 28 DME and 25 of the 121 no-DME subjects had previously received anti-VEGF therapy. Best-corrected VA was measured at a 4-meter distance using a retro-illuminated Early Treatment Diabetic Retinopathy Study (ETDRS) chart by an ophthalmic technician who was trained in the ETDRS protocol.

### 2.2. Image Acquisition

Spectral domain OCT (SDOCT) imaging of a retinal area of 20° × 15° centered on the fovea was performed using a commercially available instrument (Spectralis; Heidelberg Engineering, Heidelberg, Germany). A high-density SDOCT raster volume scan was generated from 73 raster horizontal B-scans (9 averaged frames) with a vertical spacing of 62 *μ*m. Each B-scan consisted of 1024 A-scans and had a depth resolution of 3.9 *μ*m.

### 2.3. Image Analysis

**Figure 1:**
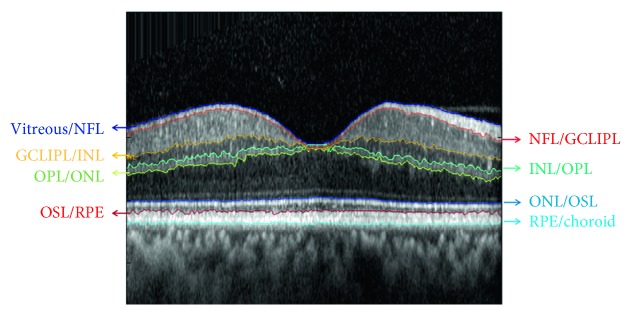
Example of an OCT B-scan in a DR subject without DME, representing the eight segmented retinal layer interfaces.

SDOCT B-scans were analyzed using our previously described automated image segmentation software based on graph theory and dynamic programming [25]. In brief, a graph was generated for all SDOCT B-scans with edge weights designated according to vertical gradients in the images. A horizontal path through the graph that minimized the total sum of the weights was found using Dijkstra's algorithm and defined a line separating two retinal cell layers.  Figure 1 displays eight retinal interfaces that were detected: (1) vitreous and NFL, (2) NFL and GCLIPL, (3) GCLIPL and INL, (4) INL and OPL, (5) OPL and outer nuclear layer (ONL), (6) ONL and OSL, (7) OSL and retinal pigment epithelium (RPE), and (8) RPE and choroid. Following our previously reported method, an operator was able to review the automated interface detection results by scrolling through all SDOCT B-scans. If necessary, errors were corrected by manually selecting the segmentation line that required adjustment and drawing a revised line corresponding to the visualized cell-layer interface. The program then regenerated an automated line by restricting the graph search area to a small vertical image region around the manually drawn line and recalculating the minimum graph cut solution, as previously described [25]. Locations of retinal layer interfaces that were not visually discernible (disruptions) due to gross abnormalities in retinal cell layer architecture were manually selected by the operator. The automated image segmentation method was previously validated [25] by demonstrating a high correlation with data provided by commercial instruments and also showing a decrease in retinal thickness with increased age, consistent with previous reports. Additionally, the average error rate obtained by the automated segmentation method was shown to be 7% in NPDR subjects [25].

Enface thickness maps and reflectance images were generated for each of 7 retinal layers (NFL, GCLIPL, INL, OPL, ONL, OSL, and RPE) based on segmentation of the 8 retinal interfaces in the SDOCT B-scans. Regions containing retinal layer interface disruption were not assigned thickness or reflectance values. Retinal layer metrics of thickness, reflectance, and areas of interface disruption were evaluated in the ETDRS central subfield (1 mm diameter) [29]. Mean thickness metrics were calculated for each layer (NFL_T_, GCLIPL_T_, INL_T_, OPL_T_, ONL_T_, and OSL_T_). Reflectance ratio metrics were also calculated in the central subfield for each layer (NFL_R_, GCLIPL_R_, INL_R_, OPL_R_, ONL_R_, and OSL_R_) as the mean intensity of each layer divided by the mean intensity of the RPE (layer_Intensity_/RPE_Intensity_).

Percent areas of layer interface disruption relative to the total central ETDRS subfield area (NFL_d_, INL_d_, ONL_d_, and RPE_d_) were calculated. NFL_d_ was calculated based on the interface disruption of both vitreous/NFL and NFL/GCLIPL interfaces relative to the total central subfield area. Similarly, INL_d_ was calculated based on disruptions of both GCLIPL/INL and INL/OPL interfaces; ONL_d_ was calculated based on disruptions of both OPL/ONL and ONL/OSL interfaces; RPE_d_ was calculated based on disruptions of both OSL/RPE and RPE/choroid interfaces.

### 2.4. Statistical Analysis

Associations of retinal layer thickness, reflectance, and percent area of interface disruptions were determined by multivariate general linear models, adjusting for age, sex, race, diabetes type, diabetes duration, and HbA1C level. Diagnostics of model assumptions were performed on all models and residuals for VA followed a normal distribution. Potential influential data points were also identified; sensitivity analyses excluding those data points were performed and the regression results were not changed materially. With a sample size of 28, the statistical power for multivariate linear regression (with 7 variables) was 80% to detect a partial correlation of 0.56 or greater, and with a sample size of 121, the power was 80% to detect a partial correlation of 0.26 or greater. For the associations between VA and retinal layer interface disruptions, Kruskal–Wallis tests were also performed and results were similar to those obtained by analysis of variance. All statistical tests were conducted using SAS 9.4 (SAS Institute Inc., Cary, NC). All *P*-values were from two-sided tests. For the associations between VA and layer-specific measurements, significance was accepted at *P* < 0.008 to account for multiple comparisons using Bonferroni correction.

## 3. Results

**Figure 2:**
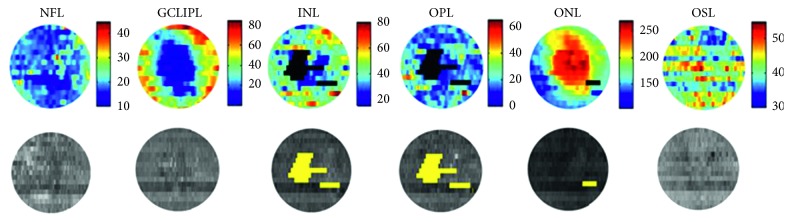
Enface thickness maps and reflectance images in a DR subject with DME. Top (left to right): ETDRS central subfield thickness maps of NFL, GCLIPL, INL, OPL, ONL, OSL, and RPE retinal layers. Bottom (left to right): reflectance images of NFL, GCLIPL, INL, OPL, ONL, OSL, and RPE retinal layers. Regions of retinal layer interface disruptions are represented by black and yellow on retinal layer thickness and reflectance maps, respectively. In these regions of disrupted interfaces, thickness and reflectance values were not assigned.

Demographic details and ocular characteristics of subjects are presented in Table 1. Mean CST in all subjects was 283 ± 56 *µ*m (*N* = 149). As expected, CST was greater in subjects with DME (371 ± 70 *µ*m; *N* = 28), compared with subjects without DME (263 ± 23 *µ*m; *N* = 121) (*P* < 0.001). Log MAR VA of all subjects was 0.04 ± 0.13. Subjects with DME had worse VA (0.14 ± 0.16 log MAR), compared with subjects with no DME (0.02 ± 0.11 log MAR) (*P* < 0.001). Examples of enface retinal layer thickness maps and reflectance images (not normalized to RPE reflectance) in a DR subject with DME are displayed in Figure 2. On average, ONL and NFL have the largest and smallest thickness, respectively. Due to the foveal depression, GCLIPL has minimal thickness in the center of the fovea. The ONL and OSL have the lowest and highest reflectance, respectively. Regions of retinal layer interface disruptions are represented by black and yellow on the thickness and reflectance layer maps, respectively.

### 3.1. Retinal Layer Thickness

Mean retinal layer thickness and regression coefficients obtained in DME subjects after adjusting for age, sex, race, diabetes type, diabetes duration, and HbA1c are listed in Table 2. Increased GCLIPL_T_ and OPL_T_ (coefficients ≥ 0.03 log MAR/10 micron) and decreased OSL_T_ (coefficient = −0.117 log MAR/10 micron) were associated with reduced VA (*P* < 0.008). That is, each 10 micron increase in GCLIPL_T_ and OPL_T_ resulted in an approximate 1 or 2 letter loss of VA, whereas each 10 micron decrease in OSL_T_ resulted in approximately 5 letters of VA loss. Mean retinal layer thickness and regression coefficients obtained in no-DME subjects after adjusting for age, sex, race, diabetes type, diabetes duration, and HbA1c are listed in Table 3. Increased INL_T_ was associated with reduced VA (coefficient = 0.048 log MAR/10 micron). Mean retinal layer thickness and regression coefficients obtained in no-DME subjects with a history of anti-VEGF treatment after adjusting for age, sex, race, diabetes type, diabetes duration, and HbA1c are listed in Table 4. Increased OPL_T_ was associated with reduced VA (coefficient = 0.061 log MAR/10 micron).

### 3.2. Retinal Layer Reflectance

Mean retinal layer reflectance and regression coefficients obtained in DME subjects after adjusting for age, sex, race, diabetes type, diabetes duration, and HbA1c are listed in Table 2. Increased NFL_R_ (coefficient = 0.077 log MAR/0.1 reflectance ratio) and decreased OSL_R_ (coefficient = −0.069 log MAR/0.1 reflectance ratio) were associated with reduced VA (*P* < 0.008). Mean retinal layer reflectance and regression coefficients obtained in no-DME subjects after adjusting for age, sex, race, diabetes type, diabetes duration, and HbA1c are listed in Table 3. Retinal layer reflectance changes were not associated with reduced VA. Similarly, in no-DME subjects with a history of anti-VEGF treatment, there was no significant association between retinal layer reflectance and VA (Table 4).

### 3.3. Retinal Interface Disruption

Both increased INL_d_ and ONL_d_ were associated with worse VA after adjusting for age, sex, race, diabetes type, diabetes duration, and HbA1c (*P* < 0.008). The mean VA values for subjects with 0%, <10%, and ≥10% INL_d_ were 0.02 ± 0.12 (*N* = 124), 0.09 ± 0.13 (*N* = 13), and 0.18 ± 0.14 (*N* = 12) log MAR, respectively (*P* < 0.01). The mean VA values in subjects with 0%, <10%, and ≥10% ONL_d_ were 0.02 ± 0.12 (*N* = 125), 0.11 ± 0.15 (*N* = 11), and 0.16 ± 0.14 (*N* = 13) log MAR, respectively (*P* < 0.01). When the analyses were conducted separately for DME and no-DME subjects, there was no significant association between VA and INL_d_ or ONL_d_ (*P* > 0.05).

## 4. Discussion

In the current study, we determined associations between VA and retinal layer thickness and reflectance in DR subjects with and without DME. In DME subjects, GCLIPL and OPL thickening and OSL thinning, as well as hyper-reflectance of the NFL and hypo-reflectance of the OSL, were associated with reduced VA. Moreover, VA was reduced in regions of disrupted retinal interfaces bordering the INL and ONL. Interestingly, in subjects without DME, thickening of the INL was associated with reduced VA, whereas in the subset of these subjects who had previous anti-VEGF treatment, thickening of OPL was associated with reduced VA.

In DME subjects, increased GCLIPL_T_ and OPL_T_ were associated with decreased VA. The relationship between increased OPL_T_ and decreased VA is consistent with previous studies, which reported a correlation between increased edema and reduced VA [20]. Our findings are also consistent with other reports of the presence of cystoid spaces or increased OPL thickness, although the relation to VA was not reported in these studies [9, 11]. Decreased OSL_T_ was associated with reduced VA, consistent with the findings of a previous study [18]. This change in OSL_T_ suggests that photoreceptor degeneration, possibly secondary to macular edema, may contribute to vision loss. Further studies are needed to confirm our findings, given the limited number of DME subjects in this study, and to determine whether treatment based on thickening of specific retinal layers can potentially improve visual outcomes.

In subjects without DME, increased INL_T_ was associated with reduced VA. This finding suggests that thickening of the INL can occur and perhaps precede the development of DME. Of note, the regression coefficient for the association between INL_T_ and VA in no-DME subjects with a history of anti-VEGF treatment was similar to the value in subjects without DME, though it did not reach statistical significance after adjusting for multiple comparisons, possibly due to the smaller sample size. Interestingly, in no-DME subjects with a history of anti-VEGF treatment, thickening of OPL was associated with reduced VA, similar to the finding in DME subjects. Future longitudinal studies are needed to investigate thickness alterations in this retinal layer as an early indicator for recurrent DME or as a marker for the adequacy of DME treatment.

The current study demonstrated associations between changes in retinal layer reflectance and reduced VA. In the DME subjects, decreased OSL_R_ and increased NFL_R_ were associated with reduced VA. The association of OSL_R_ and VA is consistent with a previous study that reported reduced reflectance of the photoreceptor layer when cystoid spaces were present in the OPL [30] and other studies that showed a correlation between the continuity of the photoreceptor inner and outer segment (ellipsoid) and VA [7, 19–21, 31, 32]. However, these previous studies evaluated the inner and outer segment junction visibility based on a single B-scan or a low-density raster of B-scans. In the current study, enface reflectance images of the OSL were obtained from high-density OCT B-scans, thereby providing a more accurate localization of the spatial extent of reduced OSL reflectance. To our knowledge, an association between increased NFL_R_ and reduced VA has not been previously reported.

Several previous studies have reported methods for segmentation of different retinal layers in DR [33–39]. The accuracy of automated segmentation of retinal layers in healthy and DR subjects relies on the presence of distinct interfaces between layers. However, in DME subjects who have severe pathology, these interfaces cannot be clearly identified, even by expert human evaluation. The method presented in the current study allowed quantitative measurement of areas in which retinal layer interfaces were visually indiscernible due to pathologies and macular edema, thus providing a useful metric for assessing retinal integrity. The finding of an association between reduced VA and disrupted INL and ONL interfaces is consistent with previous studies that reported a correlation between combined inner retinal interface disruptions (NFL/GCLIPL, GCLIPL/INL/OPL, and OPL/ONL) and VA, although they did not evaluate disruptions in other layer interfaces (ONL/OSL and OSL/RPE) [22, 23].

## 5. Conclusion

Quantitative assessment of retinal layer integrity by enface OCT imaging may be clinically relevant for monitoring the progression of pathologies due to disease or their resolution following treatment. Concurrent assessment of thickness, reflectance, and interface disruption of individual retinal layers by enface OCT imaging provides a comprehensive approach for identifying anatomic outcomes that may be useful for monitoring development, progression, and treatment efficacy of DME.

## Figures and Tables

**Table 1:** Study population and ocular characteristics.

	Total sample size = 149
*Population characteristics*	
Age, mean (SD), years	56.0 (11.8)
Female sex, number (%)	87 (58.4%)
Race/ethnicity, number (%)	
White	25 (16.8%)
African American	77 (51.7%)
Hispanic or Latino	42 (28.2%)
Asian	5 (3.4%)
DM type, number (%)	
1	12 (8.1%)
2	137 (91.9%)
DM duration, mean (SD), years	16.3 (10.1)
Glycated hemoglobin level, mean (SD), %	7.9 (1.7)

*Ocular characteristics*	
Study eye, number (%)	
Right	96 (64%)
Left	53 (36%)
Spherical equivalent refractive error, mean (SD), D	−0.66 (1.74)
Visual acuity, mean (SD), log MAR units	0.04 (0.13)
Diabetic retinopathy stage, number (%)	
No DR	51 (34%)
NPDR	59 (40%)
PDR	39 (27%)
Presence of DME, number (%)	28 (18.8%)
Central subfield thickness, mean (SD), *μ*m	283 (56)

DM = diabetes mellitus; DR = diabetic retinopathy; PDR = proliferative diabetic retinopathy; NPDR = nonproliferative diabetic retinopathy; DME = diabetic macular edema; SD = standard deviation.

**Table 2:** Multivariate associations of retinal layer thickness and reflectance ratio with logMAR visual acuity based on data from DME subjects (*N *=* *28).

Metrics	Thickness (microns), mean* *±* *SD	Regression coefficient (95% CI) [1]
NFL_T_	21* *±* *6	0.074 (−0.020, 0.167)
GCLIPL_T_	43 ± 26	0.030 (0.013, 0.048)^*∗∗*^
INL_T_	55 ± 57	0.014 (0.003, 0.024)
OPL_T_	24 ± 8	0.101 (0.043, 0.160)^*∗∗*^
ONL_T_	159 ± 57	−0.007 (−0.018, 0.003)
OSL_T_	42 ± 9	−0.117 (−0.165, −0.069)^*∗∗*^

Metrics	Reflectance ratio, mean ± SD	Regression coefficient (95% CI) [1]
NFL_R_	0.63 ± 0.12	0.077 (0.040, 0.114)^*∗∗*^
GCLIPL_R_	0.65 ± 0.07	0.085 (−0.003, 0.173)
INL_R_	0.49 ± 0.08	−0.028 (−0.123, 0.068)
OPL_R_	0.53 ± 0.08	0.057 (−0.035, 0.150)
ONL_R_	0.35 ± 0.04	0.012 (−0.130, 0.154)
OSL_R_	0.76 ± 0.11	−0.069 (−0.117, −0.021)^*∗∗*^

^*∗∗*^ *P* ≤ 0.008. ^1^Regression coefficients represent logMAR change with 10 micron increase in thickness or 0.1 increase in reflectance ratio, after adjusting for age, sex, race, diabetes type, diabetes duration, and HbA1c. NFL* *=* *nerve fiber layer; GCLIPL* *=* *ganglion cell layer* *+* *inner plexiform layer; INL* *=* *inner nuclear layer; OPL* *=* *outer plexiform layer; ONL* *=* *outer nuclear layer; OSL* *=* *photoreceptor outer segment layer; RPE* *=* *retinal pigment epithelium. Suffix T = thickness; suffix R = reflectance ratio.

**Table 3:** Multivariate associations of retinal layer thickness and reflectance ratio with logMAR visual acuity based on data from subjects without DME (*N* = 121).

Metrics	Thickness (microns), mean ± SD	Regression coefficient (95% CI) [1]
NFL_T_	18 ± l3	0.004 (−0.060, 0.068)
GCLIPL_T_	28 ± 12	−0.003 (−0.021, 0.016)
INL_T_	21 ± 8	0.048 (0.023, 0.073)^*∗∗*^
OPL_T_	21 ± 8	0.029 (0.001, 0.056)
ONL_T_	114 ± 22	−0.002 (−0.012, 0.007)
OSL_T_	44 ± 7	−0.008 (−0.036, 0.019)

Metrics	Reflectance ratio, mean ± SD	Regression coefficient (95% CI) [1]
NFL_R_	0.54 ± 0.07	0.029 (0, 0.057)
GCLIPL_R_	0.61 ± 0.07	0.021 (−0.010, 0.052)
INL_R_	0.50 ± 0.06	0.010 (−0.025, 0.044)
OPL_R_	0.52 ± 0.06	0.009 (−0.026, 0.045)
ONL_R_	0.37 ± 0.05	0.043 (0.001, 0.085)
OSL_R_	0.81 ± 0.07	−0.007 (−0.037, 0.023)

^*∗∗*^ *P* ≤ 0.008. ^1^Regression coefficients represent logMAR change with 10 micron increase in thickness or 0.1 increase in reflectance ratio, after adjusting for age, sex, race, diabetes type, diabetes duration, and HbA1c. NFL = nerve fiber layer; GCLIPL = ganglion cell layer + inner plexiform layer; INL = inner nuclear layer; OPL = outer plexiform layer; ONL = outer nuclear layer; OSL = photoreceptor outer segment layer; RPE = retinal pigment epithelium. Suffix T = thickness; suffix R = reflectance ratio.

**Table 4:** Multivariate associations of retinal layer thickness and reflectance ratio with logMAR visual acuity based on data from subjects without DME but with a history of anti-VEGF treatment (*N* = 25).

Metrics	Thickness (microns), mean ± SD	Regression coefficient (95% CI) [1]
NFL_T_	18 ± 3	0.132 (−0.054, 0.318)
GCLIPL_T_	29 ± 11	0.012 (−0.034, 0.057)
INL_T_	28 ± 10	0.046 (0.007, 0.086)
OPL_T_	21 ± 12	0.061 (0.028, 0.093)^*∗∗*^
ONL_T_	117 ± 39	−0.009 (−0.02, 0.002)
OSL_T_	41 ± 8	−0.021 (−0.078, 0.036)

Metrics	Reflectance ratio, mean ± SD	Regression coefficient (95% CI) [1]
NFL_R_	0.55 ± 0.09	0.031 (−0.037, 0.098)
GCLIPL_R_	0.61 ± 0.08	0.017 (−0.056, 0.09)
INL_R_	0.49 ± 0.07	−0.002 (−0.079, 0.076)
OPL_R_	0.51 ± 0.07	−0.014 (−0.09, 0.061)
ONL_R_	0.38 ± 0.06	0.01 (−0.068, 0.088)
OSL_R_	0.79 ± 0.07	0.003 (−0.079, 0.085)

^*∗∗*^ *P* ≤ 0.008. ^1^Regression coefficients represent logMAR change with 10 micron increase in thickness or 0.1 increase in reflectance ratio, after adjusting for age, sex, race, diabetes type, diabetes duration, and HbA1c. NFL = nerve fiber layer; GCLIPL = ganglion cell layer + inner plexiform layer; INL = inner nuclear layer; OPL = outer plexiform layer; ONL = outer nuclear layer; OSL = photoreceptor outer segment layer; RPE = retinal pigment epithelium. Suffix T = thickness; suffix R = reflectance ratio.

## Data Availability

The data used to support the findings of this study are available from the corresponding author upon request.
